# Inflammatory Crosstalk Between Type 2 Diabetes and Sarcopenia: Insights from In Silico Evaluation

**DOI:** 10.3390/ijms26167932

**Published:** 2025-08-17

**Authors:** Cristina Russo, Maria Stella Valle, Maria Teresa Cambria, Lucia Malaguarnera

**Affiliations:** 1 Section of Pathology, Department of Biomedical and Biotechnological Sciences, School of Medicine, University of Catania, 95123 Catania, Italy; lucmal@unict.it; 2 Section of Physiology, Department of Biomedical and Biotechnological Sciences, University of Catania, 95123 Catania, Italy; m.valle@unict.it; 3 Section of Biochemistry, Department of Biomedical and Biotechnological Sciences, School of Medicine, University of Catania, 95123 Catania, Italy

**Keywords:** sarcopenia, diabetes, mitochondrial dysfunction, adipokines and myokines, metabolic inflammation, transcriptomic signature

## Abstract

Sarcopenia and type 2 diabetes mellitus (T2DM) are chronic conditions that gradually affect the elderly, often coexisting and interacting in complex ways. Sarcopenia, which is characterized by the progressive loss of muscle mass and function, is frequently observed in individuals with T2DM. Although the clinical association is well known, the molecular mechanisms remain unclear. Gene expression datasets were retrieved from the Gene Expression Omnibus database. DEGs were identified using the limma package in R (R 4.4.0). Shared DEGs were subjected to Gene Ontology (GO) and Kyoto Encyclopedia of Genes and Genomes (KEGG) enrichment analyses. Protein–protein interaction networks were constructed using the STRING database and were visualized with Cytoscape. Hub genes were identified via six topological algorithms in the CytoHubba plugin. Pearson’s correlation analysis was conducted between hub genes and selected metabolic regulators. GO and KEGG enrichment analyses indicated that mitochondrial function, oxidative phosphorylation, and immune–inflammatory responses were significantly enriched. A PPI network revealed a mitochondrial hub of five key genes involved in energy metabolism, whose downregulation suggests mitochondrial dysfunction as a shared mechanism in sarcopenia and T2DM. Our results provide new insight into the molecular overlap between T2DM and sarcopenia, highlighting potential biomarkers and therapeutic targets for addressing both metabolic disruption and muscle decline.

## 1. Introduction

Type 2 diabetes mellitus (T2DM) is one of the most common chronic metabolic diseases, representing a significant health concern in the elderly population and affecting about 25% of the world’s population over the age of 65 years [1]. This percentage is likely to increase in the coming decades. Type 2 diabetes is characterized by hyperglycemia that occurs in individuals with insulin resistance (IR) and a relative insulin deficiency. In addition to the microvascular and macrovascular complications that cause comorbidities and a range of geriatric disorders, elderly people with T2DM are affected by a significant amount of functional disability [2]. Among these, sarcopenia is, more recently, included as an increasing complication, affecting about 10% to 27% of the elderly T2DM population compared to those who have normal blood glucose levels [3]. Thus, T2DM and sarcopenia, which are traditionally regarded as separate clinical entities, are significantly interconnected, particularly regarding pathophysiology and clinical outcomes [4]. Sarcopenia, which is characterized by the progressive loss of skeletal muscle mass, strength, and function, has a profound impact on mobility, independence, and overall quality of life [5,6]. Recent evidence indicates that diabetes accelerates muscle degeneration through mechanisms such as insulin resistance, mitochondrial dysfunction, oxidative stress, and chronic low-grade inflammation [7]. Inflammation plays a central role in the bidirectional relationship between these two conditions. In T2DM, adipose tissue dysfunction leads to an increased secretion of pro-inflammatory cytokines such as TNF-α, IL-6, and CRP, which not only impair insulin signaling but also contribute to muscle protein degradation and hinder muscle regeneration [8,9]. Among the molecular mediators bridging these pathological processes, chitinases have emerged as both biomarkers and potential effectors of inflammation. In diabetes, hyperglycemia and insulin resistance can trigger systemic inflammatory responses that may extend to the central nervous system. In the brain, this neuroinflammation contributes to neuronal dysfunction and neurodegeneration [10]. Similarly, in sarcopenia, muscle atrophy, mitochondrial dysfunction, and the associated infiltration of inflammatory cells exacerbate systemic inflammation, promoting insulin resistance and further impairing glycemic control [11].

This vicious cycle has led to the emergence of the concept of diabetic sarcopenia, which describes the co-occurrence of type 2 diabetes mellitus and sarcopenia in the same individual. Although no universally accepted clinical definition currently exists, the term is increasingly employed in the literature to highlight the synergistic impact of metabolic and muscle deterioration in affected patients. Understanding the shared molecular pathways between T2DM and sarcopenia, especially those related to inflammation and metabolic dysfunction, is essential for the development of predictive biomarkers, targeted therapies, and integrated intervention strategies.

In this context, we focus on specific genes that are of particular interest due to their central role in regulating metabolism, muscle function, and inflammation. Among these, FNDC5 (irisin), a myokine that has been linked to muscle metabolism and adipose tissue function, plays a crucial role in the crosstalk between skeletal muscle and metabolic tissues [12]. Similarly, ghrelin (GHRL), known for its role in appetite regulation and energy balance, has been implicated in muscle regeneration and mitochondrial function, making it highly relevant in both sarcopenia and diabetes [13]. Other genes, such as leptin (LEP) and adiponectin (ADIPOQ), are important adipokines that are involved in regulating energy metabolism and inflammation, with potential implications in muscle degradation and metabolic dysfunction [14]. Investigating the expression and interaction of these genes in the context of both sarcopenia and diabetes will provide deeper insight into the molecular mechanisms driving these diseases and may identify new therapeutic targets. In our study, the expression profiles of these hub genes were validated using independent GEO datasets. Our findings aim to bridge the gap between sarcopenia and T2DM by uncovering shared molecular mechanisms and identifying potential biomarkers, which could inform future therapeutic strategies.

## 2. Results

### 2.1. Detection of DEGs

To explore the transcriptional changes associated with sarcopenia and type 2 diabetes, we analyzed two gene expression datasets—GSE1428 and GSE16415. Both are derived from skeletal muscle tissue. This approach allowed us to investigate two different pathological conditions affecting the same tissue type, with the aim of identifying overlapping DEGs and uncovering shared biological mechanisms, particularly those related to mitochondrial energy metabolism.

The GSE1428 dataset includes skeletal muscle biopsies (vastus lateralis) obtained from healthy individuals, both young and elderly. The comparison between older (sarcopenic) and younger controls was designed to identify genes associated with age-related muscle decline and the onset of sarcopenia. In contrast, the GSE16415 dataset focuses on skeletal muscle samples from individuals with or without type 2 diabetes. Diabetic patients were either treated with diet or metformin. By comparing diabetic individuals to non-diabetic controls, the objective was to detect transcriptional alterations in skeletal muscle associated with type 2 diabetes.

Following data normalization and differential expression analysis, using thresholds of |log_2_ fold change| ≥ 0.5 and an FDR-adjusted *p*-value < 0.05, we identified 428 DEGs in the sarcopenia dataset (GSE1428), of which 199 were upregulated and 229 were downregulated. In the diabetes dataset (GSE16415), we identified 312 DEGs, including 162 upregulated and 150 downregulated genes.

To better visualize the distribution and significance of these DEGs, volcano plots were generated for each dataset, highlighting the most significantly up- and downregulated genes. Additionally, heatmaps of the top DEGs selected based on statistical significance and magnitude of expression change revealed clear clustering patterns, supporting the distinction between the experimental groups.

This comparative approach provides insight into the molecular changes occurring in skeletal muscle under different pathological conditions and offers a basis for identifying potential shared targets involved in muscle dysfunction and metabolic disease.

**Figure 1:**
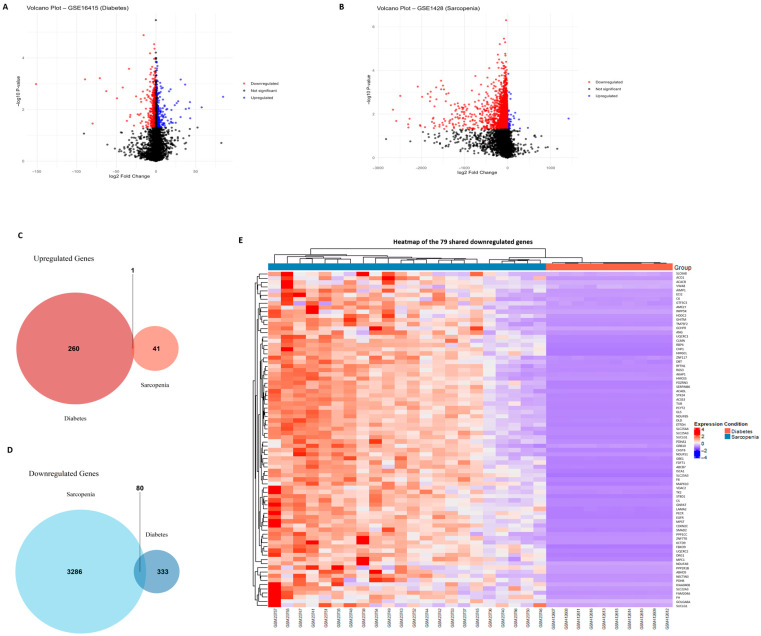
Identification of DEGs in diabetes and sarcopenia. (**A**) Volcano plot of the GSE16415 dataset (diabetes), with upregulated and downregulated DEGs highlighted similarly. (**B**) Volcano plot of the GSE1428 dataset (sarcopenia) showing significantly upregulated (red) and downregulated (blue) genes with a log_2_ fold change ≥ 0.5 and an adjusted *p*-value < 0.05. (**C**) Venn diagram showing the number of overlapping upregulated genes between the two datasets. One shared upregulated gene (NNMT) was identified. (**D**) Venn diagram illustrating the 79 downregulated DEGs shared by both sarcopenia and diabetes datasets. (**E**) Heatmap of shared DEGs between sarcopenia and diabetes datasets, illustrating consistent downregulation across conditions. Together, these visualizations support the identification of a common metabolic and mitochondrial dysfunction pattern underlying both diabetes and sarcopenia.

We also performed a Venn diagram analysis to assess the overlap of DEGs between the two datasets. This revealed 1 overlapping upregulated gene (NNMT) and 79 overlapping downregulated genes, suggesting a shared transcriptional signature across sarcopenia and diabetes (Figure 1). NNMT exhibited tissue-specific duality, whereby hepatic upregulation promoted SIRT1 stabilization, improved lipid profiles, and guarded against NAFLD progression, whereas adipose upregulation depleted methyl donors and fostered insulin resistance.

### 2.2. DEG Functional Characteristic Analysis

**Figure 2:**
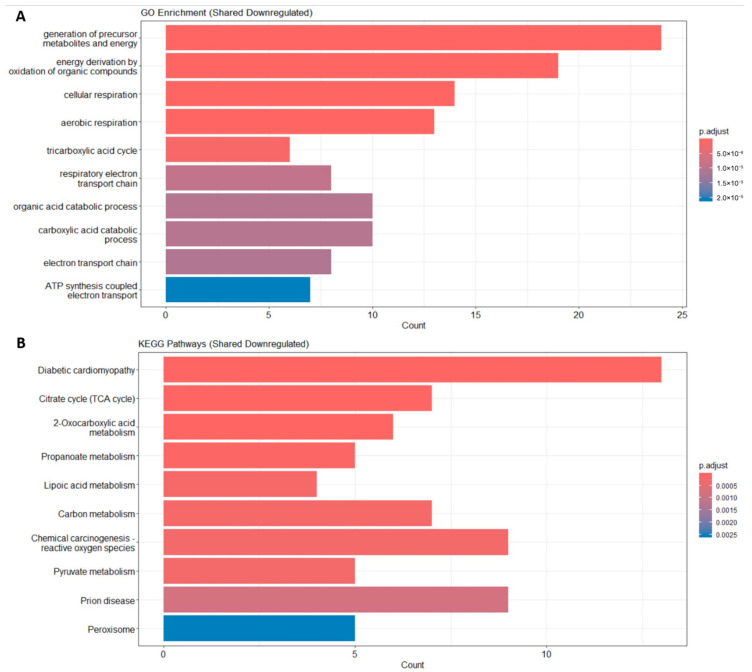
Functional enrichment. (**A**) Enrichment result of DEGs’ GO term. (**B**) Enrichment result of DEGs’ KEGG pathway.

The GO enrichment analysis of the downregulated DEGs shared between sarcopenia and diabetes revealed a significant enrichment in biological processes related to mitochondrial function and energy metabolism. Specifically, the most enriched terms included the generation of precursor metabolites and energy, cellular respiration, aerobic respiration, and the tricarboxylic acid cycle, highlighting a common mitochondrial dysfunction signature. Furthermore, processes such as ATP synthesis-coupled electron transport and the respiratory electron transport chain were significantly represented, reinforcing the hypothesis of impaired oxidative phosphorylation (Figure 2A).

Consistently, KEGG pathway analysis showed that shared downregulated DEGs were significantly associated with pathways such as diabetic cardiomyopathy, the citrate cycle, 2-oxocarboxylic acid metabolism, pyruvate metabolism, carbon metabolism, and peroxisome. These findings suggest a common metabolic impairment, particularly involving energy production and mitochondrial respiration, in both sarcopenia and diabetes (Figure 2B).

### 2.3. Construction of a PPI Network and Module Analysis

**Figure 3:**
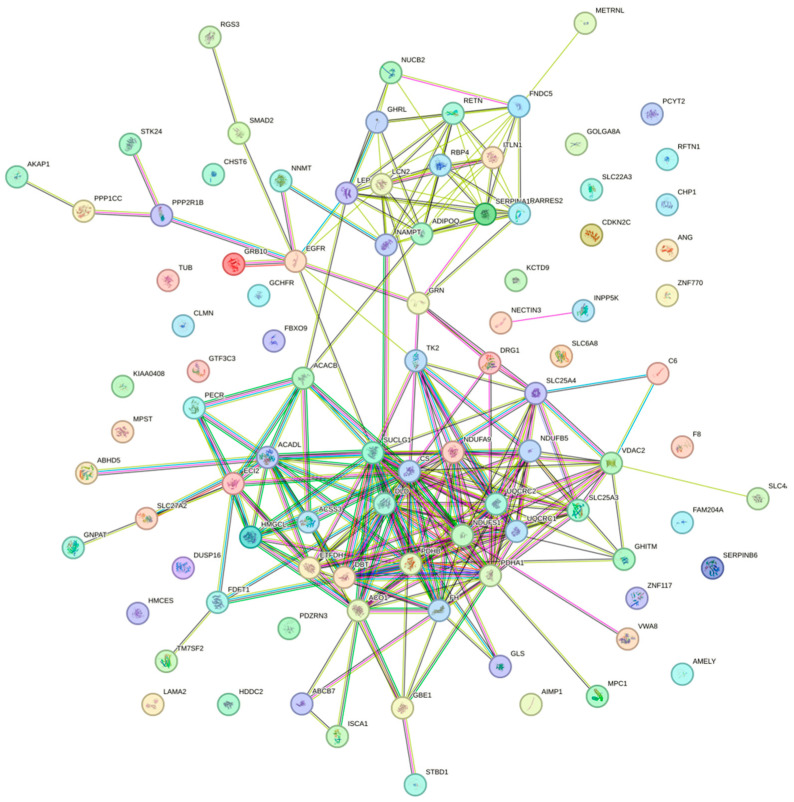
PPI network constructed using the STRING database.

To explore the potential interactions among the shared DEGs between sarcopenia and diabetes, a PPI network was constructed using the STRING database and was visualized with Cytoscape 3.10.2 software. The resulting PPI network revealed a high degree of connectivity among the shared DEGs, suggesting that these genes are functionally interrelated (Figure 3).

**Figure 4:**
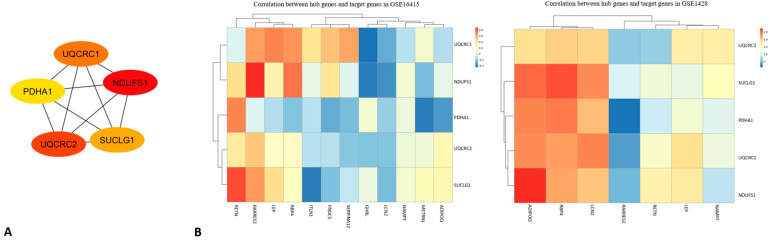
(**A**) The protein interaction network obtained by analyzing PPI with the Cytoscape plugin MCODE, representing the sub-modules obtained using the MCODE plugin. (**B**) Correlation between hub genes and target genes in GSE1428 (left) and GSE16415 (right). The heatmaps show the strength of correlations between genes, with positive correlations indicated by red and negative correlations by blue, highlighting the diverse relationships between metabolic and inflammatory genes in the datasets.

Using the MCODE plugin in Cytoscape, we identified three significant clusters (modules) within the network. The most prominent module (Cluster 1) consisted of genes primarily involved in mitochondrial energy metabolism, such as PDHA1, UQCRC1, UQCRC2, NDUFS1, and SUCLG1 (Figure 4A). These genes were mainly related to oxidative phosphorylation and the tricarboxylic acid cycle, highlighting mitochondrial dysfunction as a common feature in both sarcopenia and diabetes. In particular, PDHA1 emerged as a key mediator of the pyruvate dehydrogenase complex, linking glycolysis to the TCA cycle and supporting ATP production; it correlated positively with classical regulators EP, ITLN1, and RBP4, while correlated negatively with newer factors FNDC5, RETN, and NAMPT, reflecting its protective metabolic role. UQCRC1 and UQCRC2, which are both core subunits of mitochondrial complex III, showed strong positive associations with LEP, ITLN1, and RBP4 (UQCRC1) or LEP and ITLN1 (UQCRC2), while inversely tracking FNDC5, thereby integrating classical adipokine support and delineating the counter-regulatory influence of emerging myokines. NDUFS1, which is a complex I subunit that is essential for NADH oxidation, also aligned positively with LEP and RBP4 but was negatively regulated by FNDC5, METRNL, and RETN, highlighting its role as a nexus between energy supply and inflammatory signals. SUCLG1, which is part of succinate-CoA ligase in the TCA cycle, maintained a positive link to RBP4, which was contrasted by a consistent negative correlation with FNDC5, underscoring its function in sustaining energy homeostasis under classical adipokine control.

### 2.4. Identification and Analysis of Hub Genes

Hub genes within the PPI network were identified using the Cytoscape plugin MCODE, (Figure 4A). Five genes consistently ranked as the top hubs across different algorithms: PDHA1, UQCRC1, NDUFS1, UQCRC2, and SUCLG1. To better quantify the degree of deregulation, we assessed the fold changes in the five mitochondrial hub genes. PDHA1 was downregulated by approximately −1.6-fold in sarcopenic muscle (*p*-adjusted < 0.01) and −1.4-fold in diabetic adipose tissue (*p*-adjusted < 0.05). Similarly, UQCRC1 and UQCRC2 showed fold changes around −1.5 and −1.7, respectively, while NDUFS1 and SUCLG1 exhibited reductions of approximately −1.4-fold across both datasets. These consistent decreases in expression levels reinforce the role of mitochondrial dysfunction as a shared pathological mechanism linking sarcopenia and diabetes. These hub genes play central roles in mitochondrial energy metabolism and electron transport chain function. Their downregulation across both datasets supports the hypothesis that impaired mitochondrial bioenergetics is a critical molecular mechanism linking sarcopenia and diabetes. A functional enrichment analysis of these hub genes further confirmed their involvement in key biological processes such as ATP synthesis-coupled electron transport, oxidative phosphorylation, and the tricarboxylic acid cycle.

### 2.5. Correlation Analysis Beween Hub Genes and Metabolic Genes of Interest

Pearson’s correlation analysis between the five mitochondrial hub genes (PDHA1, UQCRC1, NDUFS1, UQCRC2, and SUCLG1) and selected metabolic genes (FNDC5, GHRL, LEP, RBP4, ADIPOQ, RETN, LCN2, etc.) revealed condition-specific associations across the two datasets (GSE1428 and GSE16415) (Figure 4B).

In the GSE1428 dataset, statistically significant positive correlations (*p* < 0.05) were observed between PDHA1 and FNDC5 (r = 0.81), UQCRC2 and LEP (r = 0.76), and NDUFS1 and ADIPOQ (r = 0.68).

These results suggest that mitochondrial genes may be co-regulated with metabolic pathways in skeletal muscle under sarcopenic conditions.

In contrast, in the GSE16415 dataset, correlations were generally weaker between SUCLG1 and RBP4 (r = 0.54, *p* = 0.07), as well as between NDUFS1 and LEP (r = 0.49, *p* = 0.09). GHRL did not show significant correlations with hub genes in either dataset (r < 0.3), suggesting a potentially independent regulatory role; this pattern, together with its known systemic actions on AMPK, GH/IGF-1, and muscle regeneration, supports the hypothesis of an indirect, systemic mode of action on muscle and energy metabolism (Figure 4B).

Among the signaling molecules, EP, ITLN1, and RBP4 uniformly supported mitochondrial hub activity, whereas FNDC5, METRNL, RETN, and NAMPT formed a counter-regulatory module that was activated under mitochondrial stress. Leptin acted as a diabetes-specific regulator of mitochondrial crosstalk, whereas ADIPOQ served that role in relation to sarcopenia, pointing to disease-tailored endocrine wiring.

Overall, mitochondrial hub genes appear more tightly associated with metabolic regulators in sarcopenia than in diabetes, reflecting enhanced transcriptional crosstalk between energy metabolism and adipokine signaling in sarcopenic muscle tissue.

As shown in the figure, two heatmaps visualize the correlation between hub genes (PDHA1, NDUFS1, UQCRC1, UQCRC2, and SUCLG1) and target genes across two different datasets (GSE1428 and GSE16415). Each cell in the heatmap represents the correlation value (ranging from −1 to 1) between a pair of genes, with color intensity varying according to the strength of the correlation (from blue for negative correlations to red for positive ones). In GSE1428 (on the left), the genes exhibit mainly weak-to-moderate correlations with each other, with some positive correlations being observed, such as between UQCRC2 and Pdha1, but these are generally not very strong. SUCLG1 shows a negative correlation with UQCRC1 and NDUFS1, suggesting an inverse relationship between these genes in this dataset. In GSE16415 (on the right), the correlations between hub genes and target genes appear more varied, with several areas showing strong (both positive and negative) correlations. UQCRC1 has a stronger positive correlation with MMP2 and LEP, while PDHA1 shows a higher correlation with ADIPOQ and RBP4. NDUFS1 exhibits a negative correlation with IL6 and TNF, suggesting a potential link to inflammatory processes in the context of diabetes or sarcopenia. The correlations suggest that hub genes are linked to various biological mechanisms across the datasets, potentially connecting energy metabolism and inflammation. Specifically, genes such as PDHA1 and UQCRC1 appear to be central in linking metabolic regulation with inflammatory and metabolic processes. These findings can be further explored to understand the interactions between metabolism and diseases such as diabetes and sarcopenia, investigating how these genes might influence inflammatory responses or metabolic regulation.

As shown in Table 1, across these hub genes, a clear pattern emerges linking protective adipokines and mitochondrial function in sarcopenic muscle, contrasted by the opposing behavior of FNDC5. UQCRC2, SUCLG1, PDHA1, and UQCRC1 each display strong positive correlations with AdipoQ and RBP4 (and LCN2 in the case of UQCRC2 and SUCLG1), underscoring a coordinated muscle–mitochondria–adipokine network that favors metabolic resilience. SUCLG1’s weaker association with the inflammatory marker RARRES2 further highlights its protective profile, while PDHA1 mirrors SUCLG1’s pattern, reinforcing the mitochondrial basis of this axis. UQCRC1 similarly exemplifies the AdipoQ–mitochondria partnership, with FNDC5 inversely tracking these markers. Notably, NDUFS1 stands apart—it lacks a strong link to RBP4 yet still opposes FNDC5, suggesting that even within this network, there are distinct regulatory nuances. Altogether, these observations point to a functional axis in which adipokines and mitochondrial genes act in concert to support muscle health, with FNDC5 being consistently regulated in the opposite direction. Moreover, the juxtaposition of positive adipokine–mitochondria correlations and widespread negative correlations with FNDC5 hints at a potential dysregulation of the mitochondrial network in relation to adipocytic hormones.

As shown in Table 2, classical adipokines and immunomodulators consistently show positive links with mitochondrial hub genes, while FNDC5 and other emerging adipokines demonstrate opposite trends. For example, UQCRC1 associates strongly with leptin (LEP), ITLN1, and RBP4 but associates inversely with FNDC5 and METRNL, illustrating this split. NDUFS1 mirrors that pattern and also opposes resistin (RETN). PDHA1 pairs only with ITLN1 on the positive side yet is negatively correlated with FNDC5, RETN, and NAMPT, hinting at a mitochondria-driven protective profile. UQCRC2 again favors LEP and ITLN1 over FNDC5, and SUCLG1’s sole positive tie to RBP4, alongside a consistent negative link to FNDC5, reinforces the theme that a coordinated classical adipokine–mitochondria axis exists in sarcopenic muscle, counterbalanced by FNDC5 and related factors.

Moreover, the data reveal a dualistic adipokine–mitochondria landscape in both sarcopenia and diabetes, whereby classical regulators EP, ITLN1, and RBP4 uniformly track positively with mitochondrial hub genes, indicating a preserved energy-support axis, whereas newer myokines/adipokines FNDC5, METRNL, RETN, and NAMPT are inversely correlated, hinting at a compensatory or counter-regulatory network. Finally, the divergent behavior of leptin positively correlated only in diabetes, while AdipoQ was only positively correlated in sarcopenia; this points to pathology-specific regulatory axes layered atop this shared network.

## 3. Discussion

This study utilizes a comprehensive bioinformatics approach to explore the molecular interplay between sarcopenia and T2DM. By leveraging transcriptomic data from the GEO database, we identified DEGs shared by the two conditions. Our analysis uncovered a set of upregulated and downregulated genes that may contribute to the pathophysiological overlap of muscle degeneration and metabolic dysregulation.

Functional enrichment analysis using GO and KEGG indicated that these shared DEGs are predominantly involved in inflammatory responses, mitochondrial function, and metabolic pathways. To further dissect the molecular landscape, we constructed a PPI network and performed module analysis using the MCODE plugin in Cytoscape. Hub genes were identified based on six ranking algorithms within the CytoHubba plugin, pinpointing key molecular nodes that may drive the crosstalk between sarcopenia and T2DM.

Transcriptomic and bioinformatics approaches offer valuable insights into the complex gene expression networks involved in both diseases. By identifying common DEGs, regulatory pathways, and hub genes, these analyses can reveal the molecular crosstalk between muscle and metabolic health, providing new avenues for therapeutic intervention.

Through an expanded integrative analysis of sarcopenia and type 2 diabetes transcriptomes, we delineate a multifaceted mitochondrial–adipokine–epigenetic network that underpins muscle wasting and metabolic dysfunction. First, common downregulated DEGs were overwhelmingly enriched in mitochondrial energy pathways such as oxidative phosphorylation, the TCA cycle, ATP-coupled electron transport, and peroxisomal fatty acid metabolism, indicating a profound collapse of cellular bioenergetics and redox balance in both conditions.

Recent studies have begun to explore the molecular intersection between sarcopenia and type 2 diabetes. In particular, Huang et al. (2022) performed a transcriptomic analysis identifying 15 shared differentially expressed genes (DEGs) and three hub genes between these two conditions [15]. However, their analysis focused mainly on general metabolic pathways without investigating the inflammatory–mitochondrial axis or the regulatory role of emerging adipokines and myokines.

Instead, our study emphasizes the centrality of mitochondrial dysfunction and highlights the differential correlation of mitochondrial hub genes with classical and novel adipokines, such as FNDC5 and meteorin-like (METRNL), providing new insights into the endocrine–metabolic crosstalk underlying both diabetes and sarcopenia.

Therefore, our results expand on previous studies investigating the common molecular features between sarcopenia and T2DM. Huang et al. identified three shared hub genes (COL1A1, COL3A1, and FN1) that were mainly involved in extracellular matrix remodeling, while our analysis highlighted five mitochondria-related hub genes (PDHA1, UQCRC1, UQCRC2, NDUFS1, and SUCLG1) that were linked to oxidative phosphorylation and energy metabolism. Furthermore, while Huang et al. did not focus on the involvement of adipokines or myokines, our study uncovers a dual regulatory network in which classical adipokines (e.g., RBP4, ITLN1, and LEP) and emergent myokines (e.g., FNDC5 and METRNL) differentially correlate with mitochondrial hub genes, suggesting novel endocrine signatures of sarcopenia and diabetes [15]. This provides a complementary and broader perspective on the shared pathophysiology of these conditions.

Mitochondrial dysfunction, as identified in this analysis, is a critical feature that is common to both sarcopenia and diabetes. PPI mapping then identified a central five-gene mitochondrial hub (PDHA1, UQCRC1, NDUFS1, UQCRC2, and SUCLG1), whose coordinated downshift likely drives diminished respiratory chain flux and ATP production, further confirming the importance of impaired mitochondrial bioenergetics in the pathology of both diseases. Additionally, tissue-specific modulation by Nicotinamide N-methyltransferase (NNMT) emerged as a key factor in the analysis. Hepatic NNMT upregulation promotes SIRT1 stabilization, lowers serum triglycerides and free fatty acids, and protects against the progression of non-alcoholic fatty liver disease (NAFLD), whereas adipose NNMT upregulation depletes methyl donors, fostering insulin resistance and the development of non-alcoholic steatohepatitis (NASH) [16].

Subsequently, correlation analysis revealed a bifurcated adipokine landscape—classical regulators EP, ITLN1, and RBP4 were observed in both datasets, plus LEP in diabetes—maintaining strong positive ties to mitochondrial hubs, signifying a conserved energy–support axis. In contrast, newer myokines/adipokines FNDC5, METRNL, RETN, and NAMPT, together with AdipoQ in sarcopenia, were uniformly negatively correlated, suggesting a counter-regulatory or compensatory circuit activated by mitochondrial stress. FNDC5’s inverse relationship across both pathologies suggests a shared adaptive mechanism to restore mitochondrial function under muscle-wasting or hyperglycemic stress, potentially mediated by irisin upregulation, whereas RBP4’s persistent positive association underscores its pivotal role in orchestrating mitochondrial substrate flux. The divergence of LEP (diabetes-specific) versus AdipoQ (sarcopenia-specific) further emphasizes distinct endocrine wiring that tailors adipokine–mitochondria crosstalk to each disease’s metabolic context. Of note, although GHRL exhibited only modest negative associations with hub genes in diabetes, reflecting its systemic rather than local transcriptomic mode of action, its known roles in AMPK activation, GH/IGF-1 modulation, and muscle regeneration warrant deeper exploration of inter-tissue signaling axes.

These observations also provide a foundation for investigating how systemic hormones, like ghrelin, could modulate mitochondrial function in both muscle and metabolic tissues, potentially serving as an indirect yet powerful regulator of mitochondrial and muscle metabolism. It should be noted that the associations reported here are purely correlational and do not imply causal relationships or direct regulatory effects. These findings should therefore be interpreted as statistical associations that warrant further experimental investigation.

Mechanistically, the consistent downregulation of mitochondrial genes across diabetes and sarcopenia may result from chronic oxidative stress, impaired mitochondrial biogenesis, and persistent low-grade inflammation—hallmarks of both aging and metabolic diseases. One plausible upstream regulator is PGC-1α, which is a master transcriptional coactivator of mitochondrial biogenesis, whose expression is known to decline in aged and diabetic skeletal muscle [17]. Moreover, inflammatory cytokines such as TNF-α and IL-6, which are elevated in both T2DM and sarcopenia, have been shown to suppress mitochondrial function by inhibiting PGC-1α and inducing mitochondrial damage [18]. These factors could jointly drive the transcriptional repression of key mitochondrial genes (e.g., PDHA1, UQCRC1, and NDUFS1), ultimately compromising energy metabolism and muscle function.

Collectively, this panoramic view articulates a core mitochondrial dysfunction signature common to sarcopenia and diabetes, a classical adipokine–mitochondria support network, a novel counter-regulatory myokine/adipokine module, and tissue- and disease-specific modifiers (NNMT, LEP, and AdipoQ). This integrated framework offers multiple therapeutic intervention nodes, ranging from NNMT modulation and SIRT1 activation to the fine-tuning of classical versus emerging adipokines. These interventions could restore mitochondrial health, rebalance energy metabolism, and ultimately ameliorate both muscle atrophy and metabolic derangement. Importantly, targeting key metabolic and inflammatory pathways, such as those regulated by RBP4, FNDC5, and AdipoQ, may help develop novel therapeutic strategies to combat the underlying mitochondrial dysfunction in these conditions. While the classical adipokines (e.g., leptin and RBP4) maintain consistent positive correlations with mitochondrial hubs across both diseases, newer adipokines and myokines like FNDC5 and METRNL appear to form a counter-regulatory module that is activated during mitochondrial stress. The inverse regulation of FNDC5 in both sarcopenia and diabetes suggests its role as part of an adaptive mechanism to counteract mitochondrial dysfunction, particularly under conditions of muscle-wasting or metabolic stress. Additionally, the tissue-specific expression of adipokines such as AdipoQ in sarcopenia and LEP in diabetes further underscores the pathology-specific regulatory mechanisms that modulate mitochondrial function through endocrine signaling. These findings reveal how distinct adipokine networks are activated depending on the disease context, highlighting potential biomarkers for disease diagnosis and prognosis. From a clinical perspective, genes such as RBP4 and FNDC5 could serve as promising biomarkers for the early detection and monitoring of metabolic–muscular dysfunction in both sarcopenia and T2DM. Moreover, their dual involvement in energy metabolism and endocrine signaling suggests they could be explored as potential therapeutic targets, for example, by modulating adipokine–mitochondria crosstalk through pharmacological or lifestyle interventions. Future experimental investigations are required in order to confirm the expression patterns of the identified hub genes in independent cohorts using experimental approaches in order to validate this in silico analysis and to develop innovative therapeutic strategies targeting mitochondrial biogenesis, which can restore muscle and metabolic health in these populations (Table 3).

## 4. Materials and Methods

### 4.1. Data Collection

We searched for gene expression datasets related to diabetes and sarcopenia using keywords in the GEO database National Center for Biotechnology Information (NCBI), Bethesda, MD, USA (https://www.ncbi.nlm.nih.gov/geo/, accessed on 11 August 2025) [19]. The GEO database, created and maintained by the National Center for Biotechnology Information (Bethesda, MD, USA), hosts high-throughput gene expression and microarray data submitted by research organizations worldwide. The goal of our search was to identify datasets that could provide valuable insights into gene expression patterns associated with diabetes, sarcopenia, and inflammation.

Based on our specified inclusion criteria, two microarray datasets were downloaded: GSE1428 and GSE16415. These datasets were generated using different Affymetrix platforms: GPL96 [HG-U133A] Affymetrix Human Genome U133A Array for GSE1428, and GPL570 Affymetrix Human Genome U133 Plus 2.0 Array for GSE16415.

The GSE1428 dataset focuses on the global gene expression profile of the vastus lateralis muscle in 10 young male subjects (aged 19–25 years) and 12 older male subjects (aged 70–80 years). In contrast, the GSE16415 dataset includes data from 9 lean individuals and 39 obese patients. It examines differential gene expression in visceral adipose tissue from Asian Indian women with type 2 diabetes mellitus, compared to age- and BMI-matched healthy controls with normal glucose tolerance. Genome-wide transcriptomic profiling was performed on 5 diabetic and 5 control subjects.

By analyzing these two datasets, we can gain valuable insights into gene expression changes associated with sarcopenia, as well as molecular differences between control and diabetic individuals.

### 4.2. Identification of DEGs

To identify DEGs, we analyzed two publicly available microarray datasets from the Gene Expression Omnibus (GEO) database: GSE1428 and GSE16415. GSE1428 includes skeletal muscle samples (vastus lateralis) from young healthy adults and elderly individuals with age-related sarcopenia, while GSE16415 comprises muscle biopsies from type 2 diabetic patients and matched non-diabetic controls. The datasets were generated using the Affymetrix Human Genome U133A Array (GPL96) and the Affymetrix Human Genome U133 Plus 2.0 Array (GPL570), respectively. Expression data were normalized and processed using the limma package in R. To account for the use of different microarray platforms (GPL96 and GPL570), cross-platform normalization was performed using the ComBat function from the sva R package(R 4.4.0), as well as standard background correction and log_2_ transformation that were implemented in the limma pipeline. This approach minimizes batch effects and ensures comparability between datasets. Multiple testing correction was applied using the Benjamini–Hochberg false discovery rate (FDR) method to reduce the likelihood of false positives. A linear model was fitted and contrasts between groups were defined. DEGs were selected based on adjusted *p*-value < 0.05 and absolute log_2_ fold change (|log_2_FC|) ≥ 0.5. Venn diagrams were then used to identify shared DEGs between sarcopenia and diabetes.

### 4.3. Enrichment Analyses of DEGs

Gene Ontology (GO) and Kyoto Encyclopedia of Genes and Genomes (KEGG) enrichment analyses were performed to explore the biological functions and pathways associated with the DEGs. The lists of significantly upregulated and downregulated DEGs from GSE1428 and GSE16415 were first intersected to identify shared genes. Functional enrichment analysis was carried out using the cluster Profiler package in R, with the org.Hs.eg.db database. GO enrichment was performed for Biological Process (BP), while KEGG was used to identify enriched signaling pathways. Dot plots were generated using the enrichplot package to visualize the most significant terms (*p* adjusted < 0.05).

### 4.4. PPI Network Construction and Module Analysis

To investigate the potential interactions among the shared DEGs, we constructed a PPI network using the Search Tool for the Retrieval of Interacting Genes/Proteins (STRING), Swiss Institute of Bioinformatics (SIB), Lausanne, Switzerland (https://string-db.org/, accessed on 18 July 2025 [20]. Gene symbols of shared DEGs were submitted with a minimum interaction confidence score of 0.4. The resulting interaction network was exported and visualized in Cytoscape (v3.10.0). A modular analysis of the PPI network was performed using the MCODE plugin in Cytoscape. The MCODE parameters were set as follows: K-core = 2, degree cutoff = 2, maximum depth = 100, and node score cutoff = 0.2. The genes within each module (cluster) were subjected to functional enrichment analysis using the same GO and KEGG pipeline described above, before being visualized in R.

### 4.5. Identification and Evaluation of Hub Genes

To identify central genes within the network, we used the CytoHubba plugin in Cytoscape. Hub genes were identified by applying six ranking algorithms: Degree, Maximal Clique Centrality (MCC), Maximum Neighborhood Component (MNC), Closeness, Radiality, and EcCentricity. The top 10 genes from each algorithm were intersected using the online tool Venny 2.1 Venny 2.1, BioinfoGP, Centro Nacional de Biotecnología (CNB-CSIC), Madrid, Spain (https://bioinfogp.cnb.csic.es/tools/venny/, accessed on 18 July 2025). The common genes across algorithms were selected as hub genes. Furthermore, GeneMANIA (http://www.genemania.org, accessed on 18 July 2025) was used to construct a co-expression and functional interaction network to evaluate the hub genes in a broader biological context [21].

### 4.6. Correlation Analysis Between Hub Genes and Metabolic Genes of Interest

A Pearson correlation analysis was conducted to explore potential regulatory relationships between identified hub genes and metabolically relevant genes. Five hub genes (PDHA1, UQCRC1, NDUFS1, UQCRC2, and SUCLG1), previously selected based on the PPI network, were correlated with a curated list of metabolic genes, including FNDC5, GHRL, LEP, ADIPOQ, RARRES2, RETN, NAMPT, LCN2, ITLN1, RBP4, and SERPINA12. Gene expression matrices from GSE1428 and GSE16415 datasets were used. Probe IDs were mapped to official gene symbols based on the corresponding GPL annotation files. Pearson’s correlation coefficients were computed for each pair of hub and metabolic genes. Correlation matrices were visualized as heatmaps using hierarchical clustering, allowing for a comparison of expression patterns across conditions, as well as the identification of highly correlated gene pairs.

## 5. Conclusions

**Figure 5:**
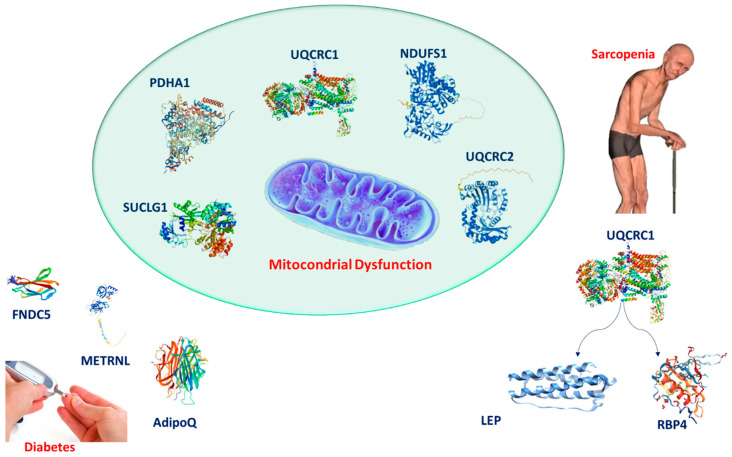
Diagram illustrating the relationship between mitochondrial dysfunction, adipokines, myokines, and their impact on sarcopenia and type 2 diabetes.

The intricate mitochondrial–adipokine–myokine network (Figure 5) highlights the central role of mitochondrial dysfunction in both sarcopenia and type 2 diabetes. Mitochondria are interconnected with various classical and novel adipokines and myokines, reflecting the complex metabolic and inflammatory responses characteristic of these conditions. These impairments contribute to muscle wasting, reduced energy production, and the exacerbation of metabolic dysfunctions. The network reveals potential therapeutic targets, from mitochondrial support to the modulation of adipokines and myokines, which could help ameliorate both muscle atrophy and metabolic derangement. This framework emphasizes the broader impact of mitochondrial health on muscle and metabolic systems and provides a clear direction for future research. Furthermore, the complex interplay between mitochondrial dysfunction, adipokine signaling, and inflammation offers numerous avenues for further investigation. Future studies should focus on identifying the molecular pathways that govern mitochondrial–adipokine interactions, particularly the modulation of emerging adipokines like FNDC5 and METRNL. Understanding how these myokines influence mitochondrial function and metabolic health could offer new insights into the pathophysiology of sarcopenia and diabetes, leading to more effective therapeutic strategies. Additionally, exploring the systemic role of hormones like ghrelin and their indirect effects on mitochondrial function opens the door for novel inter-tissue signaling mechanisms that could provide broader therapeutic benefits.

## Figures and Tables

**Table 1:** Correlation analysis of hub genes in muscle tissue, highlighting their positive and negative associations with key metabolites and inflammatory markers.

*Hub Gene*	*Positive Correlation*	*Negative Correlation*	*Observation*
***UQCRC2***	*RBP4,* *LCN2*	*FNDC5,* *NAMPT*	*RBP4 and LCN2 are positively associated with UQCRC2 in sarcopenic muscle, while FNDC5 exhibits a strong inverse correlation, suggesting the existence of a functional muscle–mitochondria–adipokine axis.*
***SUCLG1***	*AdipoQ,* *RBP4,* *LCN2*	*RARRES2*	*SUCLG1 is strongly associated with AdipoQ and RBP4 but shows weaker links to inflammatory markers (RARRES2).*
***PDHA1***	*AdipoQ,* *RBP4*	*FNDC5*	*PDHA1 follows a similar pattern to SUCLG1, indicating a mitochondria-linked metabolic profile with protective potential.*
***UQCRC1***	*AdipoQ,* *RBP4*	*FNDC5*	*AdipoQ and mitochondria work together to maintain healthy muscle metabolism. FNDC5 behaves in the opposite manner—when AdipoQ and mitochondrial markers increase, FNDC5 decreases, and* vice versa.
***NDUFS1***	*AdipoQ*	*FNDC5,* *LCN2*	*It is the only factor lacking strong correlations with RBP4, yet it still opposes FNDC5.*

**Table 2:** Correlation analysis of hub genes in relation to adipokines and metabolic markers.

*Hub Gene*	*Positive Correlation*	*Negative Correlation*	*Observation*
***UQCRC1***	*LEP, ITLN1, RBP4*	*FNDC5, METRNL*	*Mitochondrion associated with leptin and ITLN1 but were inversely related to FNDC5 and METRNL.*
***NDUFS1***	*LEP, RBP4*	*FNDC5, METRNL, RETN*	*Similar pattern to UQCRC1, but also inversely related to RETN.*
***PDHA1***	*ITLN1*	*FNDC5, RETN, NAMPT*	*Shows a strong negative correlation with inflammatory/metabolic markers (RETN, NAMPT).*
***UQCRC2***	*LEP, ITLN1*	*FNDC5*	*Clear separation between classical adipokines (LEP) and novel ones (FNDC5).*
***SUCLG1***	*RBP4*	*FNDC5*	*FNDC5 is consistently negatively correlated.*

**Table 3:** Mitochondrial correlation patterns and inferred functional roles of metabolic genes and adipokines.

*Gene/Factor*	*Correlation Pattern*	*Inferred Role/Function*
*PDHA1*	*Positive with EP, ITLN1, RBP4; negative with FNDC5, RETN, NAMPT*	*Central to pyruvate dehydrogenase complex; links glycolysis to TCA cycle and supports ATP production; protective metabolic profile.*
*UQCRC1*	*Positive with LEP, ITLN1, RBP4; negative with FNDC5, METRNL*	*Core complex III subunit; integrates classical adipokine signals to maintain respiratory chain flux.*
*NDUFS1*	*Positive with LEP, RBP4; negative with FNDC5, METRNL, RETN*	*Complex I subunit; mediates NADH oxidation and mitochondrial energy supply; subject to counter-regulation by newer adipokines.*
*UQCRC2*	*Positive with LEP, ITLN1; negative with FNDC5*	*Complex III subunit; delineates separation between classical adipokine support and FNDC5’s inverse regulation.*
*SUCLG1*	*Positive with RBP4; negative with FNDC5*	*Succinate-CoA ligase subunit; supports TCA cycle flux and energy homeostasis, opposed by FNDC5.*
*NNMT*	*Tissue-specific: in liver (supports SIRT1, improves lipid profile); in adipose (methyl depletion, insulin resistance)*	*Modulates NAD^+^ metabolism and epigenetic methylation; beneficial in liver, detrimental in adipose.*
*EP*	*Positive with mitochondrial hubs*	*Classical metabolic regulator; supports mitochondrial energy production.*
*ITLN1*	*Positive with mitochondrial hubs*	*Immunomodulator and metabolic regulator; promotes mitochondrial function.*
*RBP4*	*Positive with mitochondrial hubs*	*Retinol transporter; central mediator of mitochondrial energy handling.*
*FNDC5*	*Negative with mitochondrial hubs*	*Myokine (irisin); counter-regulatory/compensatory role under mitochondrial stress.*
*METRNL*	*Negative with mitochondrial hubs*	*Emerging myokine; involved in counter-regulation of energy metabolism.*
*RETN*	*Negative with mitochondrial hubs*	*Resistin; pro-inflammatory adipokine, inversely linked to mitochondrial function.*
*NAMPT*	*Negative with mitochondrial hubs*	*NAD biosynthesis enzyme; its inverse correlation suggests complex metabolic feedback.*
*LEP*	*Positive in diabetes only*	*Leptin; disease-specific regulator of mitochondrial–adipokine crosstalk in diabetes.*
*AdipoQ*	*Positive in sarcopenia only*	*Adiponectin; disease-specific regulator enhancing mitochondrial metabolism in sarcopenia.*
*GHRL*	*Weak/moderate negative in diabetes*	*Ghrelin; endocrine regulator with indirect effects on mitochondrial and muscle metabolism.*

## Data Availability

The original contributions presented in this study are included in the article. Further inquiries can be directed to the corresponding authors.
